# Clinical indications for antibiotic prescribing in Danish general practice

**DOI:** 10.1080/02813432.2025.2546415

**Published:** 2025-08-21

**Authors:** Zarghuna Ahengar, Christina Hovmark Pedersen, Alina Zalounina Falborg, Malene Plejdrup Hansen

**Affiliations:** a Center for General Practice at Aalborg University, Aalborg, Denmark; b Research Unit for General Practice, Aarhus, Denmark; c Department of Public Health, Research Unit for General Practice, University of Southern Denmark, Odense, Denmark

**Keywords:** Antibiotics, prescriptions, clinical indications, general practice, community-acquired infections

## Abstract

**Background:**

An updated overview of the antibiotic prescribing pattern in Danish general practice is needed to help inform continued efforts for rational antibiotic prescribing.

**Objective:**

To investigate clinical indications for antibiotic prescriptions issued in general practice in Denmark.

**Materials and methods:**

This register-based study included all redeemed antibiotic prescriptions issued in Danish general practice between 1 January 2023 and 31 December 2023. Data were extracted from ‘Antibiotikastatistik’, a publicly available register maintained by the Danish Health Data Authority. Descriptive statistics were used to analyze the distribution of the clinical indications. Furthermore, the distribution of the antibiotics prescriptions was analyzed by age across different clinical indication groups, stratified by gender.

**Results:**

A total of 1,916,910 antibiotic prescriptions were issued from Danish general practice in 2023. More than half of these prescriptions were used for treatment of either a respiratory tract infection (28.4%) or a urinary tract infection (26.7%). Throat infection and pneumonia comprised about 70% of indications for treatment of a respiratory tract infection. Prophylactic treatment was mainly used for elderly patients – and most often for urinary tract infections. Some 23.3% of the prescriptions either contained an ‘unspecific indication’ or had no indication stated.

**Conclusions:**

This study provides a solid overview of indications used for antibiotic prescriptions in Danish general practice. This information might be used for development of future antibiotic stewardship interventions.

## Introduction

Antibiotics are an important part of modern medicine, and without them we will not be able to treat common infections and perform life-saving procedures [1,2]. However, any antibiotic consumption drives the selection of resistant bacteria [3]. Worldwide antimicrobial resistance is a rapidly growing problem, and the World Health Organization (WHO) has designated antimicrobial resistance as one of the top global health threats [1].

In Denmark, about 90% of human antibiotic use originates from the primary healthcare sector [4] – with the majority (about 75%) prescribed within the general practice setting [5].

Since 2011, the overall consumption of antibiotics in Denmark has been steady decreasing, mainly due to a reduction in antibiotic use within the primary healthcare sector [6]. However, in 2023, a 6.6% increase in total antibiotic use in the primary healthcare sector was observed [6].

When Danish doctors issue an antibiotic prescription, they are legally required to provide a clinical indication [7]. The indication must be documented in the patient’s electronic medical record, known as the ‘Fælles Medicin Kort’ (FMK), and in the patient’s journal [8]. In 2009, a nationwide electronic prescription system was introduced in Denmark to record these indications. The system was fully implemented in 2011, enabling GPs to specify a clinical indication for the prescription either from a predefined drop-down menu or by entering free text [9]. By documenting clinical indications for antibiotic prescriptions, it has become possible to systematically monitor prescribing patterns.

A previous Danish study from general practice – based on prescription data from 2013 – identified that most antibiotic prescriptions were issued for patients diagnosed with either a respiratory tract infection or a urinary tract infection [9]. Importantly, it was also found that a rather large number of the issued prescriptions (17%) were labeled with a non-specific indication, for example, ‘infection’, and about a third of prescriptions had no indication provided [9]. To continue efforts for rational antibiotic prescribing – and develop new interventions for antimicrobial stewardship – it is important to have updated information about the antibiotic prescribing pattern in Danish general practice available.

This study aimed to investigate clinical indications for antibiotic prescriptions issued in general practice in Denmark from 1 January 2023 to 31 December 2023. Moreover, the distribution of antibiotic prescriptions according to age across different clinical indications and gender groups was investigated.

## Materials and methods

### Data sources

This register-based study included all redeemed antibiotic prescriptions issued in Danish general practice between 1 January 2023 and 31 December 2023. Data were extracted from ‘Antibiotikastatistik’, a publicly available register maintained by the Danish Health Data Authority [10]. The register includes prescriptions with WHO’s Anatomical Therapeutic Chemical Classification System (ATC) codes J01 (antibacterials for systemic use) or P01AB01 (metronidazole). Only prescriptions issued by doctors working in general practice were included in this present study, i.e. antibiotic prescriptions issued by dermatovenereologists, dentists, otolaryngologists, hospital physicians or by ‘other or unknown’ physicians were excluded from the dataset. Patient age and gender were reported for each antibiotic prescription.

### Clinical indications

Forty different clinical indications were provided in the dataset.

Some indications were already modified by the Danish Health Data Authority before publishing the register [10]. For example, the indication ‘urinary tract infection’ was expanded to include sub-indications such as ‘for urinary tract infection’, ‘for pyelonephritis’ and ‘for cystitis’. The ‘unknown’ category covered indications for rare diseases (e.g. Crohn’s disease), no indication reported and indications reported using free text or on paper or via telephone [10].

Before further data analysis, the authors of this present study categorized all clinical indications provided in the register – according to anatomical site: (1) respiratory tract infection (throat infection, pneumonia, otitis media, sinusitis, bronchitis and acute exacerbation of chronic bronchitis, exacerbation of chronic obstructive pulmonary disease, pertussis and other respiratory infections including lung infection treatment in cystic fibrosis), (2) skin infections (skin and soft tissue infections and erysipelas, acne, borrelia infection and other skin infections such as rosacea and staphylococci including eradication of carrier states), (3) gastrointestinal infections (peptic ulcer (eradication of *Helicobacter pylori*), diarrhea and other gastrointestinal infections including inflammation of the abdominal cavity), (4) urinary tract infections, (5) urogenital infections (chlamydia/mycoplasma infection, vaginitis inflammation of the epididymis, pelvic inflammatory disease and other urogenital infections including gonorrhea, syphilis, inflammation of the prostate gland, urethritis and chronic urinary tract infection), (6) prophylactic treatment (prophylactic treatment for urinary tract infections and other prophylactic treatment for conditions including endocarditis, severe infections, malaria or during surgery), (7) other infections (bacterial infection in bones and joints, mastitis, animal or human bites, dental, oral and jaw infection), (8) unspecified infections (infection and other unspecified infections including severe infection) and (9) missing indication (unknown).

Indications accounting for ≤0.1% of the total number of prescriptions were categorized as ‘other’.

### Data analysis

Descriptive statistics were used to analyze the distribution of the clinical indications for the issued antibiotic prescriptions. Furthermore, the distribution of the issued antibiotics prescriptions was analyzed by age across different clinical indication groups, stratified by gender.

The following age groups were used: <5 years, 5–14 years, 15–29 years, 30–49 years, 50–69 years and ≥70 years [9,11]. This distribution was chosen as differences in the antibiotic prescribing pattern within these age groups have previously been shown [9].

The results were presented as the number of prescriptions issued (*N*) and as percentages (%). Data were extracted from Excel (Redmond, WA) and analyzed in STATA version 18 (StataCorp, College Station, TX) [12].

### Ethics

The dataset is publicly available and only contains fully anonymized data.

## Results

A total of 1,916,910 redeemed antibiotic prescriptions issued from Danish general practice in 2023 were included in the analysis. Of these, 62.2% were issued for female patients (data not shown).

### Clinical indications for antibiotic prescriptions

Table 1 shows the distribution of indications used for the antibiotic prescriptions. The two most commonly used indications were: respiratory tract infections (28.4%) and urinary tract infections (26.7%). The unspecific labeling ‘against infection’ was used for 18.1% of prescriptions. A minor part of antibiotic prescriptions did not have an indication stated (5.2%).

**Table 1.** Overview of clinical indications for antibiotic prescriptions issued by Danish general practitioners in 2023.

	Antibiotic prescriptions	
Clinical indication	*N*	%
Respiratory infections		
Throat infection	216,135	11.3
Pneumonia	159,340	8.3
Otitis media	75,180	3.9
Sinusitis	59,790	3.1
Bronchitis and acute exacerbation of chronic bronchitis	23,595	1.2
Exacerbation of chronic obstructive pulmonary disease (COPD)	6135	0.3
Pertussis	3680	0.2
Other respiratory infections	65	<0.1
Total	543,920	28.4
Urinary tract infection		
Urinary tract infections	511,020	26.7
Unspecified infections		
Infection and severe infection	347,675	18.1
Skin infections		
Skin and soft tissue infections and erysipelas	219,625	11.5
Acne	35,110	1.8
Borrelia infection	23,010	1.2
Other skin infections	2530	0.1
Total	280,275	14.6
Missing indication		
Unknown	98,835	5.2
Urogenital infections		
Chlamydia/mycoplasma infection	30,555	1.6
Vaginitis	13,565	0.7
Inflammation of the epididymis	6865	0.4
Pelvic inflammatory disease	4105	0.2
Other urogenital infections	4400	0.2
Total	59,490	3.1
Prophylactic treatment		
Prophylactic for urinary tract infections	40,400	2.1
Other prophylactic treatment	5700	0.3
Total	46,100	2.4
Gastrointestinal infections		
Peptic ulcer (eradication of *Helicobacter pylori*)	9365	0.5
Diarrhea	5250	0.3
Other gastrointestinal infections	490	<0.1
Total	15,105	0.8
Other infections		
Animal or human bites	6630	0.3
Bacterial infection in bones and joints	3565	0.2
Other	4295	0.2
Total	14,490	0.8
Total	1,916,910	100

Figure 1(a–e) illustrates the distribution of indications within each of the indication categories. For respiratory tract infections, most prescriptions were prescribed for ‘throat infection’ (39.7%) or ‘pneumonia’ (29.3%) (Figure 1(a)). Almost 80% of prescriptions issued for skin infections were labeled ‘Skin and soft tissue infections and erysipelas’ (Figure 1(b)). About half of urogenital infection prescriptions were for ‘chlamydia/mycoplasma infection’ (Figure 1(c)). ‘Prophylactic for urinary tract infections’ was by far the most frequently used indication when prophylactic treatment was indicated at the prescription (87.6%) (Figure 1(d)).

### Clinical indication by age and gender

**Figure 2.**
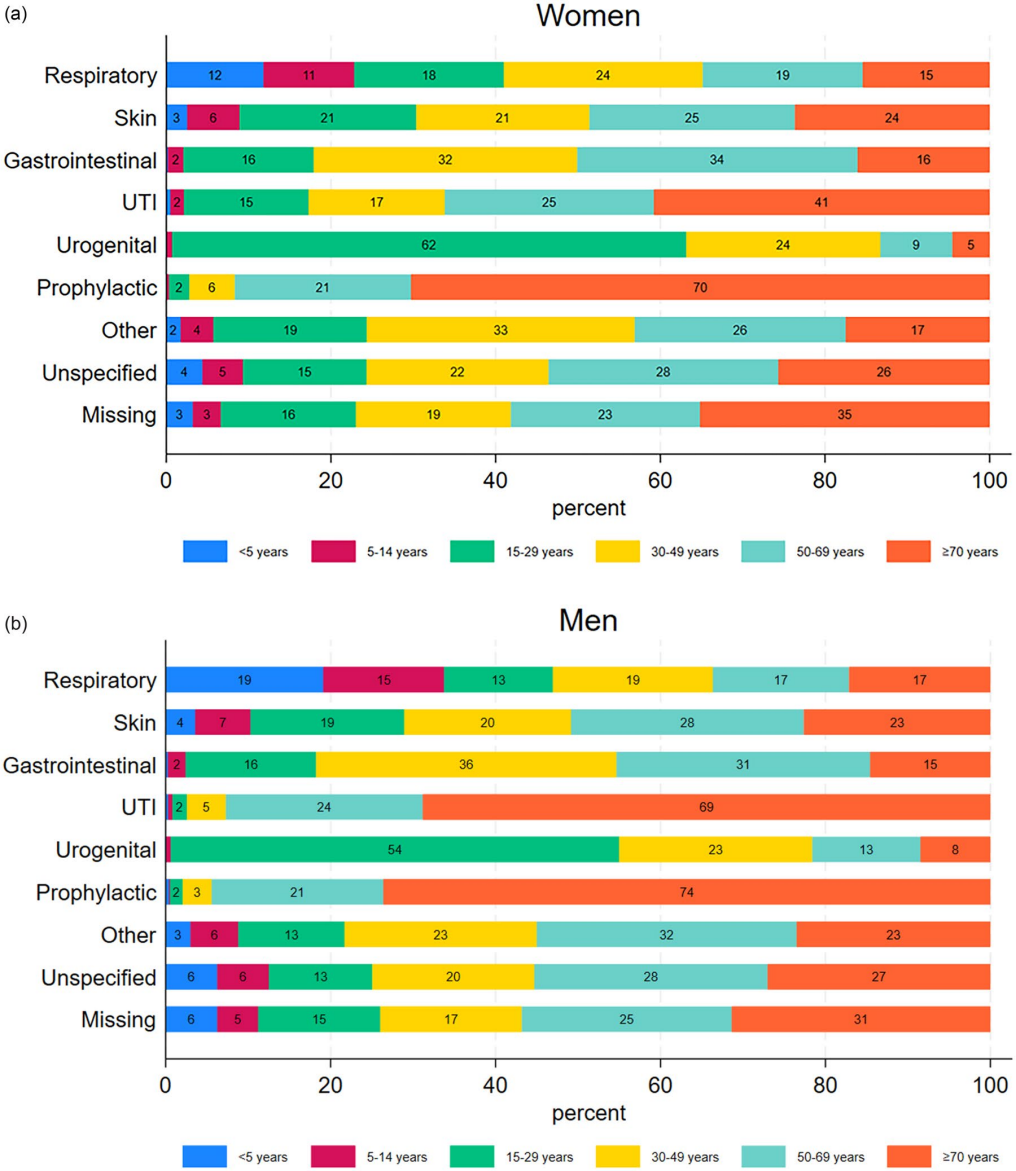
Overview of the distribution of redeemed prescriptions within each clinical indication in relation to age for (a) women and (b) men.

Figure 2(a,b) illustrates distributions of antibiotic prescriptions within clinical indications in relation to age and gender. Antibiotic prescriptions issued for a respiratory tract infection varied from 11% being issued for girls aged 5–14 years to 24% for women aged 30–49 years (Figure 2(a)). Boys aged ≤14 years accounted for a relatively large proportion of prescriptions issued for a respiratory tract infection (34%) (Figure 2(b)).

Most prescriptions issued for a urinary tract infection were used for patients aged 15 years and older – with female patients having treatment in all age groups, although a large percentage was used for women aged +50 years (66%). Men aged ≥70 years had most of the prescriptions issued for a urinary tract infection. Urogenital infection prescriptions peaked at ages 15–29 for both women (62%) and men (54%). Most prophylactic antibiotic treatments were given to patients aged ≥70 years – including both male (74%) and female patients (70%).

## Discussion

### Main findings

More than half of antibiotic prescriptions from Danish general practice were issued for patients diagnosed with either a respiratory tract infection or a urinary tract infection.

Prophylactic treatment was mainly used for elderly patients – and most often for urinary tract infections. Almost one out of four of the prescriptions either contained an ‘unspecific indication’ or had no indication stated.

Figure 1.Distribution of indications across clinical indication categories: (a) respiratory infections, (b) skin infections, (c) urogenital infections, (d) prophylactic treatment and (e) gastrointestinal infections.

**Figure:**
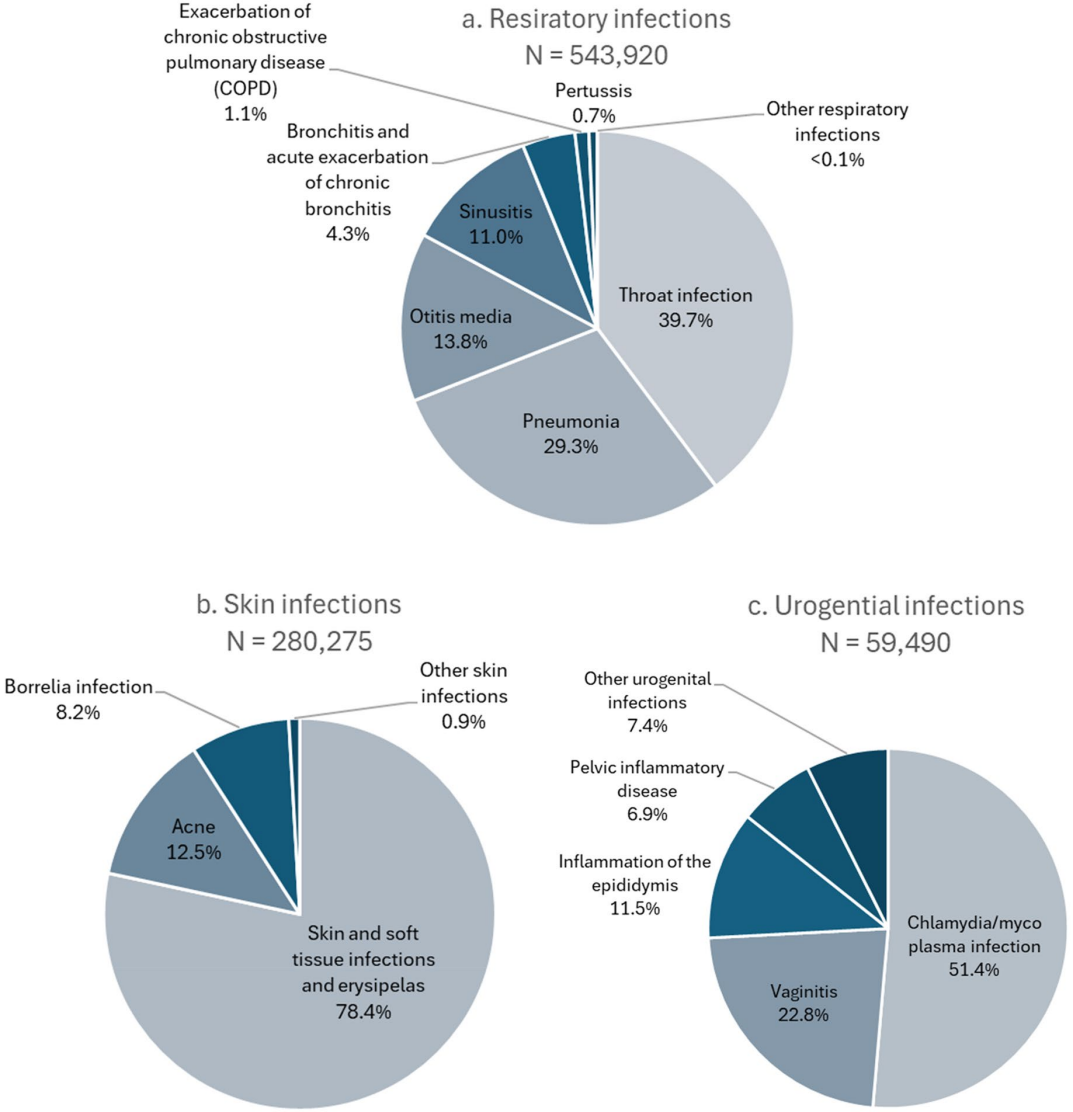

**Figure:**
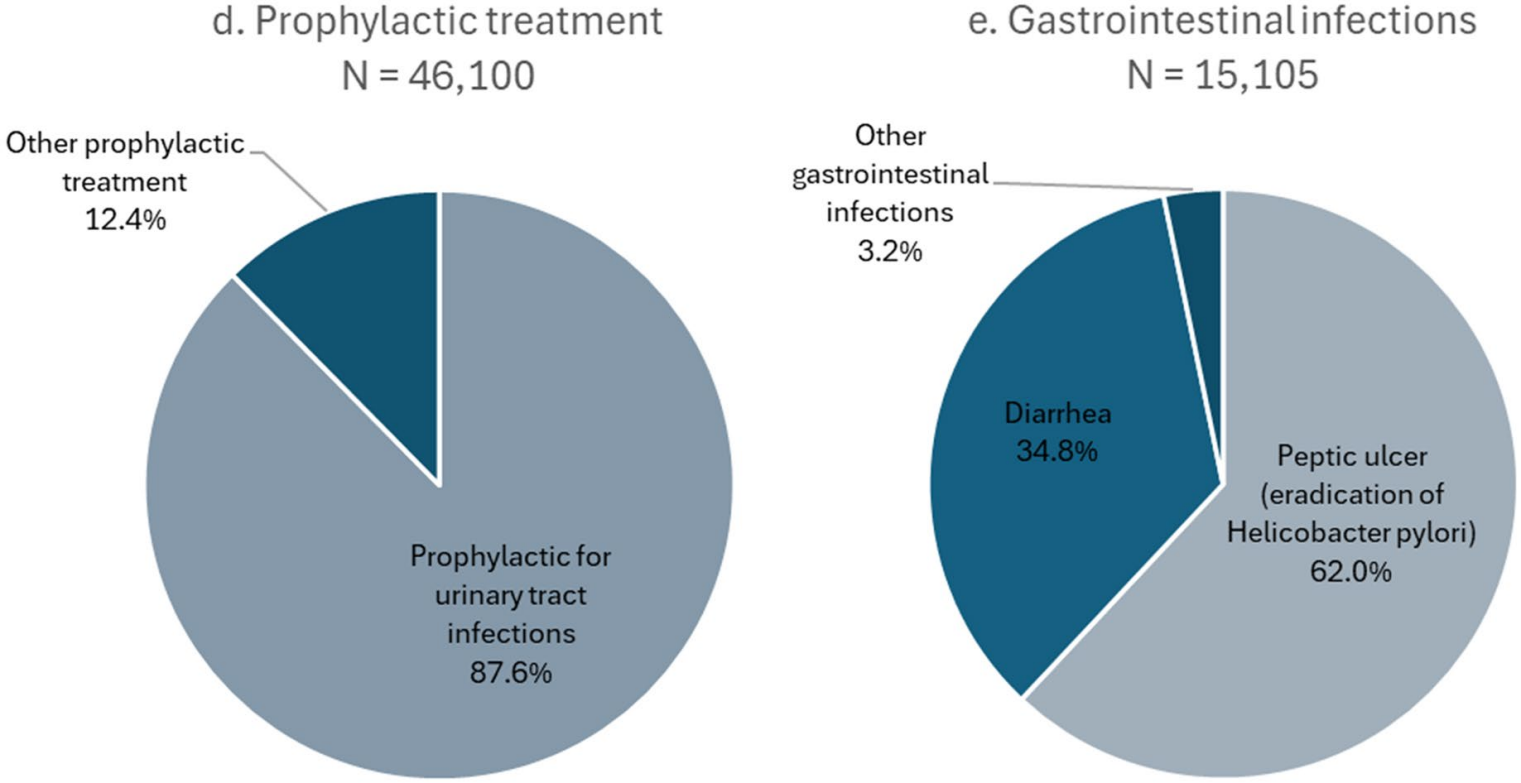

### Strengths and limitations

The study is a register-based study and includes all redeemed antibiotic prescriptions issued in general practice in Denmark. The large sample size with nearly two million prescriptions enhances the accuracy of the findings of the study. Also, the study is based on data collected from the Danish National Prescriptions Registry, which is a registry of high quality and validity [13]. However, some considerations must be acknowledged when interpreting the findings. This present study provides an insight into antibiotic dispensing, but no information on actual consumption. Interestingly, a previous Danish study from 2014 found that around 6.5% of patients being prescribed an antibiotic did not initiate treatment (primary non-adherence) [14]. Additionally, secondary non-adherence might also be considered, as it is unclear whether the patients complete the entire antibiotic course [14].

Further, the study is limited by the adjustments made in the dataset to provide a better overview of the indications provided at the antibiotic prescriptions. Indication categories such as for example, ‘urinary tract infection’ included several sub-indications such as ‘against urinary tract infection’, ‘against pyelonephritis’ and ‘against cystitis’. This categorization could potentially impact and reduce the specificity of the clinical indications presented. Moreover, indications available in the drop-down menu for antibiotic prescriptions do not fully match the International Classification of primary Care, 2nd edition (ICPC-2) codes used for coding diagnoses in Danish general practice [15]. Consequently, the risk of not obtaining the precise indication for antibiotic treatment is possible.

About 5% of the antibiotic prescriptions did not have an indication reported. Some of these missing indications could be due to handwritten prescriptions, use of free text option, or if prescriptions were issued via telephone [10]. However, as decided by the provider of this publicly available ‘Antibiotikastatistik’ dataset, the category ‘unknown’ also includes less commonly used indications (e.g. Crohn’s disease). Consequently, the true number of antibiotic prescriptions with a missing indication is expectedly lower than the percentage found in this study.

### Comparison with existing literature

The findings of our study are consistent with those reported in existing literature, including the study by Aabenhus et al. [9], which identified two major clinical indications for antibiotic prescriptions: respiratory- and urinary tract infections.

Our study, along with existing literature, has demonstrated that antibiotics for respiratory tract infections constitute a significant proportion of prescriptions issued in Danish general practice [9,11,16]. Several factors may contribute to this, but the high consumption underpins the relevance of examining whether the prescribing patterns are rational. As a result, the literature has repeatedly highlighted how acute respiratory tract infections are often treated unnecessarily with antibiotics, as these infections are frequently caused by virus for which antibiotics are ineffective [16–18]. This further emphasizes the importance of thorough investigation of prescribing patterns for the various types of acute respiratory tract infection, especially considering that every time antibiotics are used, selection of resistant bacteria occurs [19]. Notably, a Norwegian study has demonstrated that antibiotics are often prescribed for viral infections [20].

A previous Danish study identified pneumonia as the most common indication for antibiotic use for treatment of acute respiratory tract infections [11]. In our study – using year 2023 data – throat infection was the most often used indication, followed by pneumonia. This finding is consistent with the observed surge in group A streptococcal infections during the years after the COVID-19 pandemic [21]. In Denmark, both non-invasive infections, such as for example throat infection, and invasive infections, like meningitis and bacteremia, increased significantly during late 2022 and 2023 [22].

The findings of this study document an improvement in stating the clinical indications on antibiotic prescriptions. Only, 5.2% of the prescriptions issued in Danish general practice during 2023 had no indication indicated. Ten years ago, about one-third of antibiotic prescriptions had a missing indication [9]. This is a very positive trend, as knowledge of indications for antibiotic prescriptions helps direct interventions for a continued strive for rational antibiotic prescribing. Similarly, a Swedish study has found that the number of prescriptions without a diagnosis decreased from 22% in 2008 to 13% in 2013 [23].

The study by Aabenhus et al. found that 17% of the issued prescriptions were labeled with the unspecified indication ‘infection’ [9]. Similarly, we found that 18.1% of the prescriptions were categorized with this nonspecific labeling. Obviously, there may be several reasons for choosing the unspecific label ‘infection’, such as for example not being completely sure of the specific location/indication for treatment or not wanting to state specific diagnoses on the script. However, we argue that prescribers should endeavor to indicate a specific clinical indication, when possible, to obtain detailed knowledge of the antibiotic prescribing patterns.

About two-thirds of antibiotic prescriptions were issued for women, confirming previous findings indicating that women are more frequently prescribed antibiotics than males [5,11, 24,25]. A possible explanation could be that 26.7% of the prescriptions were issued for an acute urinary tract infection, which is a clinical condition that is more common in women [26]. Moreover, urinary tract infections were also found to be the most commonly used indication when providing patients with prophylactic antibiotic treatment. An interesting finding, given that antibiotic treatment to prevent recurrent urinary tract infections is generally not recommended [27]. Differences were also observed in the distributions of age across the various indications for antibiotic treatment. We found that prescriptions for urinary tract infections among men were mainly issued for men aged ≥70 years. Moreover, most patients having prophylactic treatment were above the age of 50 years. The findings suggest that the elderly generation receives a high amount of antibiotics, which is consistent with findings reported in previous studies [9,11, 28–30].

Our study found that about half of the prescriptions for urogenital infections were issued for treatment of chlamydia/mycoplasma infection – and most were issued for men and women aged 15–29 years. These findings are consistent with existing literature, which also recognizes chlamydia/mycoplasma infections as common causes of urogenital infections [31,32].

## Implications

A rational antibiotic prescribing pattern among GPs is crucial to ensure that problems with antimicrobial resistance do not escalate further. This study provides updated information on clinical indications used for antibiotic prescriptions issued in Danish general practice. More than half of antibiotic prescriptions issued in Danish general practice in 2023 were used for treatment of either a respiratory- or urinary tract infection. The results might be considered when future national antimicrobial stewardship programs are developed – although this study does not provide any knowledge of the quality of treatments, but only the quantity of antibiotics prescribed.

Importantly, we found that the number of antibiotic prescriptions with a non-specific clinical indication remains relatively high – at almost 20%. An approach to minimize this number could involve reviewing the dropdown menu, when choosing a clinical indication for an antibiotic prescription, and perhaps even deleting the non-specific label ‘against infection’, when issuing an antibiotic prescription. Also, to better understand the reasons behind the continued use of non-specific indications, future studies could investigate the decision-making processes of prescribers through interviews or surveys.
